# Correlation Between Perceived Stress Scores and Menstrual Characteristics in Young Indian Women

**DOI:** 10.7759/cureus.82921

**Published:** 2025-04-24

**Authors:** Shibu S Awasthi

**Affiliations:** 1 Physiology Department, Dr. Kailash Narayan Singh (KNS) Memorial Institute of Medical Sciences, Barabanki, IND

**Keywords:** dysmenorrhea, menstrual characteristics, menstrual irregularity, perceived stress scale-14 (pss-14), pictorial blood assessment chart (pbac)

## Abstract

Introduction: Stress is a common causative factor of menstrual irregularities, and the effect is supposed to be mediated by an alteration in normal pulsatile gonadotropin-releasing hormone (GnRH) secretion by stress.

Methodology: A cohort of 150 apparently healthy young women as volunteers was selected randomly. They were asked to attempt the perceived stress scale-14 (PSS-14) questionnaire, and based upon their scores, 50 women were allocated into group A (score of ≤28) and group B (score of ≥29), by a stratified sampling method. Their menstrual characteristics, including age of menarche, cycle length, duration of menses, any history of heavy menstrual flow (staining/passage of clots), severe debilitating dysmenorrhea, irregularities, and pictorial blood assessment chart (PBAC) scores, were recorded. The chi-square (χ^2^) test, Student's t-test, and Pearson's correlation coefficient were used to analyze the data using SPSS software version 21.0 (IBM Corp., Armonk, NY).

Results: Group B had higher PBAC scores than group A (110.24±70.00 versus 87.56±38.51; t=2.007 and p=0.047). A history of menstrual irregularity was more common in group B as compared to group A (22% versus 4%; χ^2^=7.162 and p=0.002), and a history of heavy menstrual flow was also more common in group B as compared to group A (72% versus 44%; χ^2^=8.046 and p=0.005). In the overall selected population, PSS scores were positively correlated with a history of heavy menstrual flow (r=0.267; p=0.007) and a history of debilitating dysmenorrhea (r=0.246; p=0.014). PBAC scores were positively correlated with menstrual irregularity (r=0.497; p<0.001), the duration of menses (r=0.422; p<0.001), a history of debilitating dysmenorrhea (r=0.212; p=0.034), and a history of heavy menstrual flow (r=0.212; p=0.034). The duration of menses was positively correlated with menstrual cycle length (r=0.287; p=0.004) and a history of debilitating dysmenorrhea (r=0.211; p=0.035). The history of heavy menstrual flow was also positively correlated with the history of debilitating dysmenorrhea (r=0.323; p=0.001).

Conclusion: Women suffering from higher perceived stress have higher menstrual blood flow, greater blood loss, and more chances of menstrual irregularities. Perceived stress levels are positively correlated with a history of dysmenorrhea and heavy menstrual flow. Menstrual blood loss is more in women with menstrual irregularities, longer menstrual duration, a history of dysmenorrhea, and heavy menstrual flow. Longer menstrual cycles tend to have longer menstrual duration. Dysmenorrhea is positively correlated with heavier menstrual flow.

## Introduction

Perceived stress is the subjective feeling or thoughts that an individual has about how stressed they are at a given point in time or over a given time period. Individuals might suffer from similar negative events in life, but they might appraise the impact or severity of these events to different extents. This occurs as a result of various factors such as individual personality, coping resources, and peer support [1].

The perceived stress scale-14 (PSS-14) is a Likert-like scaling questionnaire comprising 14 questions with seven positive questions and seven negative questions. PSS scores are then obtained by reversing responses (e.g., 0=4, 1=3, 2=2, 3=1, and 4=0) to the seven positively stated items and then summating across all scale items. Possible total scores range from 0 to 56. A higher score indicates greater perceived stress. It is the most widely used questionnaire for the assessment of perceived stress [1], and furthermore, it has been proved that higher PSS scores are associated with higher levels of cortisol, a biological indicator of stress [2].

Studies have shown that chronic stress causes a reduction in gonadotropin-releasing hormone (GnRH) pulsations and hence reduced luteinizing hormone (LH) secretion, whereas acute stressors can transiently increase gonadal steroid secretion [3].

Menstrual characteristics such as cycle length, menstrual duration, cycle regularity, menstrual blood loss, menstrual blood flow, and the degree of menstrual discomfort are under the direct influence of ovarian hormonal secretion throughout the menstrual cycle [4].

Pictorial blood assessment chart (PBAC) is an established and reliable tool for the assessment of menstrual blood loss. During the course of the menstrual period, the use of tampons and sanitary pads is recorded by placing a tally mark under the day next to the box. It represents how heavily stained the sanitary materials were each time they were changed. Any incidence of cloth soiling/clot passage is recorded by placing a tally mark in the clots/flooding row under the relevant day [5]. The scoring system is explained in detail in the methodology.

The PBAC cutoff score for menorrhagia was found to be "100" with a good sensitivity of 88% and a specificity of 97% [6]. We hypothesize that higher perceived stress has an adverse impact on various menstrual characteristics.

## Materials and methods

A pilot prospective cohort study was planned in which 150 young apparently healthy women were enrolled on a volunteer basis. Subjects were selected among relatives of patients visiting the general medicine OPD of King George's Medical University (KGMU), Lucknow city, Uttar Pradesh state, India. Their age group ranged from 18 to 25 years, and their ethnicity was Southeast Asian (Indian nationality). The study was commenced after obtaining ethical clearance from the King George's Medical University Institutional Ethics Committee (approval number, 724/Ethics/19; reference code, 96th ECM 11 B-Thesis/p34). The subjects were excluded if they had a history of a diagnosed gynecological disorder, any diagnosed psychiatric illness, any endocrine abnormality, features suggestive of obesity or malnourishment, or any chronic systemic illness.

Anthropometric data with subject particulars were recorded, and written consent was obtained from each subject. They were asked to attempt the PSS-14 questionnaire. Among the subjects, 88 had PSS scores of ≥29, and 62 women had PSS-14 scores of ≤28. Being a pilot study, the sample size was chosen as 100 subjects. The subjects were reassigned in the study by the method of stratified sampling, with 50 members in group A (PSS-14 scores of ≤28) and 50 members in group B (PSS-14 scores of ≥29), to eliminate selection bias. Confounders such as lifestyle differences and dietary diversity were eliminated during the enrolment process.

The subjects were followed through for one month each, and their menstrual history of that month was recorded, comprising cycle length, menstrual duration, any history of heavy menstrual flow (staining/passage of clots), severe debilitating dysmenorrhea, and menstrual irregularities [7,8]. Their age of menarche was also recorded. They were also asked to fill out their PBAC forms, which is an indirect yet accurate measure of total menstrual blood. The scoring of the PBAC questionnaire was done as depicted in Table 1 [5].

**Table 1:** PBAC scoring system The total score is obtained by summing up individual day scores for a particular menstrual episode PBAC: pictorial blood assessment chart

Pictorial depiction of menstrual blood loss	Score
Lightly stained pad/tampon	1 point
Moderately stained pad/tampon	5 points
Saturated pad	20 points
Saturated tampon	10 points
5-cent-sized clot passage	1 point
50-cent-sized clot passage	5 points
Episode of flooding/soiling of clothes	5 points

Their individual data were tabulated. The statistical analysis was done using the Statistical Product and Service Solutions software (SPSS, version 21.0; IBM SPSS Statistics for Windows, Armonk, NY). Data were presented in the form of mean±standard deviation and percentages. It was analyzed using the chi-square (χ^2^) test, Student's t-test, and Pearson's correlation coefficient.

## Results

Women suffering from higher stress levels (group B) had higher PBAC scores than women under lower stress (group A) (110.24±70.00 versus 87.56±38.51; t=2.007 and p=0.047) (Table 2).

**Table 2:** Comparison of the menstrual profile of group B and group A subjects (quantitative/numerical variables) Student's t-test has been employed with t values mentioned; p values of <0.05 are considered significant *Demarcation of significance PBAC, pictorial blood assessment chart; SD, standard deviation

Serial number	Menstrual profile	Group B (n=50)		Group A (n=50)		Significance of differences	
		Mean	SD	Mean	SD	"t" value	"p" value
1	Age at menarche (years)	13.12	±1.24	13.12	±0.95	0.000	1.000
	Range	10-16 years		11-15 years			
2	Span of menstrual cycle (days)	29.16	±2.40	29.22	±1.99	0.134	0.893
	Range	21-35 days		24-34 days			
3	Duration of menses (days)	4.88	±1.29	4.58	±0.94	1.315	0.191
	Range	3-8 days		3-7 days			
4	PBAC score	110.24±70.00		87.56±38.51		2.007	0.047*
	Range	30-404		25-159			

A history of menstrual irregularity was significantly more in group B as compared to group A (22% {n=11} versus 4% {n=2}; χ^2^=7.162 and p=0.002), and a history of heavy menstrual flow was also more common in group B as compared to group A (72% {n=36} versus 44% {n=22}; χ^2^=8.046 and p=0.005) (Table 3).

**Table 3:** Comparison of the menstrual profile of group B and group A subjects (qualitative/categorical variables) The chi-square test has been employed with chi-square values mentioned; p values of <0.05 are considered significant *Demarcation of significance

Serial number	Menstrual profile	Group B (n=50)		Group A (n=50)		Significance of differences	
		Number of subjects	Percentage of total subjects (%)	Number of subjects	Percentage of total subjects (%)	Chi-square value	"p" value
1	History of irregular menstrual cycle	11	22.0%	2	4.0%	7.162	0.002*
2	History of clots or staining	36	72.0%	22	44.0%	8.046	0.005*
3	History of severe discomfort	24	48.0%	16	32.0%	2.667	0.102

In the overall selected population, PSS scores were positively correlated with a history of heavy menstrual flow (r=0.267; p=0.007) and a history of debilitating dysmenorrhea (r=0.246; p=0.014). PBAC scores were positively correlated with menstrual irregularity (r=0.497; p<0.001), the duration of menses (r=0.422; p<0.001), a history of debilitating dysmenorrhea (r=0.212; p=0.034), and a history of heavy menstrual flow (r=0.212; p=0.034). The duration of menses was positively correlated with menstrual cycle length (r=0.287; p=0.004) and a history of debilitating dysmenorrhea (r=0.211; p=0.035). The history of heavy menstrual flow was also positively correlated with the history of debilitating dysmenorrhea (r=0.323; p=0.001) (Table 4).

**Table 4:** Correlations among PSS-14, PBAC, and menstrual parameters in the overall population "r"=Pearson's correlation coefficient; negative (-) "r" value indicates inverse correlation; p values of <0.05 are considered significant Level of correlation: *weak, **mild, and ***moderate PSS, perceived stress scale; PBAC, pictorial blood assessment chart; ≈, sign of correlation

Serial number	Correlation parameters	"r" values	"p" value
1	PSS score ≈ menarche	-0.098	0.331
2	PSS score ≈ irregularity	0.162	0.108
3	PSS score ≈ menstrual cycle span	0.016	0.874
4	PSS score ≈ menses duration	0.111	0.270
5	PSS score ≈ PBAC	0.179	0.079
6	PSS score ≈ history of clot/stain	0.267*	0.007
7	PSS score ≈ history of dysmenorrhea	0.246*	0.014
8	Menarche ≈ menstrual Irregularity	0.146	0.147
9	Menarche ≈ menstrual cycle span	-0.038	0.708
10	Menarche ≈ menses duration	-0133	0.187
11	Menarche ≈ history of clot/stain	0.001	0.994
12	Menarche ≈ history of dysmenorrhea	0.004	0.971
13	Menarche ≈ PBAC score	0.064	0.527
14	Menstrual irregularity ≈ menstrual cycle span	0.074	0.461
15	Menstrual irregularity ≈ menses duration	0.196	0.051
16	Menstrual irregularity ≈ history of clot/stain	0.148	0.141
17	Menstrual irregularity ≈ history of dysmenorrhea	0.109	0.279
18	Menstrual irregularity ≈ PBAC Score	0.497**	<0.001
19	Menstrual cycle span ≈ menses duration	0.287*	0.004
20	Menstrual cycle span ≈ history of clot/stain	-0.046	0.649
21	Menstrual cycle span ≈ history of dysmenorrhea	-0.052	0.609
22	Menstrual cycle span ≈ PBAC score	0.102	0.314
23	Menses duration ≈ history of clot/stain	0.154	0.126
24	Menses duration ≈ history of dysmenorrhea	0.211*	0.035
25	Menses duration ≈ PBAC score	0.422**	<0.001
26	History of clot/stain ≈ history of dysmenorrhea	0.323**	0.001
27	History of clot/stain ≈ PBAC score	0.212*	0.034
28	History of dysmenorrhea ≈ PBAC score	0.212*	0.034

## Discussion

Stress is a factor known to influence the characteristics of the menstrual cycle deleteriously. According to Breen et al., there exists a cortisol-mediated suppression of gonadotropin secretion [9]. This negative influence of cortisol may be modulated by sex steroid hormones, and kisspeptin signalling has also been implicated in the process [10]. Decreased hypothalamic Kiss1 mRNA expression has been observed during exposure to stress. The role of kisspeptin in mediating stress inputs is further supported by the fact that the expression of the glucocorticoid receptor is found on murine kisspeptin neurons [11]. Hypothalamic corticotropin-releasing hormone (CRH) neurons, which are important regulators of the stress response, can also directly modulate GnRH excitability in a dose-dependent and receptor-specific manner, and the GnRH response to CRH is also influenced by estrogens [12]. However, little was known about the relationship between perceptual stress in the quantified aspect and its direct effect on menstrual health.

Our study demonstrated a significant elevation of PBAC scores, representing the amount of menstrual blood loss, in women experiencing higher perceived stress scores. However, any direct correlation between perceived stress scores could not be established with PBAC scores in the overall study population. Also, we demonstrated a significant difference between our groups as group B women suffered more with heavy menstrual flow. This finding is in contrast to Nagma et al. who could not establish any significant difference in PBAC scores and menstrual flow in women having higher PSS scores and lower PSS scores [13]. Also, they employed the PSS-10 version instead of PSS-14. However, similar to our study, they also demonstrated a correlation of PSS scores with menstrual irregularity. No other relevant study has been done to our knowledge in relation to the above parameters.

Other studies conducted by Yamamoto et al. [14] and Bae et al. [15] also correlated higher perceived stress with menstrual irregularity. However, a study conducted by Fenster et al. contradicted this finding [16].

According to studies conducted by Singh et al. [17], Jung et al. [18], Rafique and Al-Sheikh [19], and Ekpenyong et al. [20], higher stress levels were positively correlated with menorrhagia, the passage of clots, and dysmenorrhea, which is consistent with our results.

Our study also demonstrated a significant positive correlation between greater menstrual blood loss (PBAC scores) and menstrual irregularity, heavier menstrual flow, and menses duration. We have also found a significant positive correlation of menses duration with a history of dysmenorrhea and menstrual cycle span and a history of heavy menstrual flow (staining/passage of clots) with a history of dysmenorrhea. No studies in our knowledge have been known to obtain such a correlation.

A plausible cause for this abnormal hypothalamic-pituitary-gonadal (HPG) axis regulation is that, in response to the perception of stress, there is subsequent CRH and cortisol release, which influences the kisspeptin-neurokinin B-dynorphin neurons (KNDy) and hence disturbs sex steroid homeostasis. Although stress seems to be an influential cause of menstrual irregularities and disorders witnessed in the study, possible confounders such as narrow age group, socio-economic similarities, and similar dietary habits also affect menstrual health.

Our study has a major limitation in that, being a pilot study, a small sample size was considered, and further research is necessary in this field of interest. Also, further studies require the employment of the measurement of levels of cortisol and sex steroids, which may augment our knowledge in depth.

However, the stratified simple sampling method has been employed in the process of selecting subjects, yet a potential bias is that women selected in this study were obtained from a single tertiary-level medical center of excellence, that is, relatives of patients in the general medicine OPD of KGMU, Lucknow. A multicentric study could eliminate this bias in future studies.

This pioneering study paves the path for further research upon PBAC scores in various menstrual health conditions and the employment of PBAC scores in gynecological health assessment, keeping in mind the role of day-to-day stressors, which can influence menstrual health. Also, it can be understood that lifestyle modifications can improve the menstrual health of society.

## Conclusions

We conclude from the knowledge gained by our study that women suffering from higher perceived stress (PSS-14 scores) also suffer higher menstrual blood flow and greater blood loss (PBAC scores), and they also have more chances of menstrual irregularities. Perceived stress scale (PSS) scores are positively correlated with a history of debilitating dysmenorrhea and heavier menstrual flow. Menstrual blood loss is quantitatively more in women with menstrual irregularities, longer menstrual duration, and a positive history of debilitating dysmenorrhea and those experiencing heavier menstrual flow. Longer menstrual cycles also tend to have a longer menstrual duration. Debilitating dysmenorrhea is more common with heavier menstrual flow.
